# Cortical excitability stratifies neurochemical profiles in amyotrophic lateral sclerosis

**DOI:** 10.1093/braincomms/fcag292

**Published:** 2026-07-28

**Authors:** Sicong Tu, Colin J Mahoney, William Huynh, Steve Vucic, Matthew C Kiernan

**Affiliations:** Brain and Mind Centre, The University of Sydney, Sydney, NSW 2050, Australia; Sydney Medical School, Faculty of Medicine & Health, The University of Sydney, Sydney, NSW 2006, Australia; Neuroscience Research Australia, Scientia Professor of Neuroscience, University of NSW, Sydney, NSW 2031, Australia; Brain and Mind Centre, The University of Sydney, Sydney, NSW 2050, Australia; Sydney Medical School, Faculty of Medicine & Health, The University of Sydney, Sydney, NSW 2006, Australia; Department of Neurology, Liverpool Hospital, South-Western Sydney Local Health District, Sydney, NSW 2170, Australia; Brain and Mind Centre, The University of Sydney, Sydney, NSW 2050, Australia; Sydney Medical School, Faculty of Medicine & Health, The University of Sydney, Sydney, NSW 2006, Australia; Brain and Nerve Research Centre, Concord Clinical School, The University of Sydney, Sydney, NSW 2139, Australia; Neuroscience Research Australia, Scientia Professor of Neuroscience, University of NSW, Sydney, NSW 2031, Australia

**Keywords:** amyotrophic lateral sclerosis, motor neuron disease, magnetic resonance spectroscopy, cortical excitability, threshold-tracking TMS

## Abstract

Transsynaptic deficits arising from an imbalance in excitatory/inhibitory inter-neuronal circuitry have been extensively shown to underlie the phenomena of altered cortical motor excitability in patients with amyotrophic lateral sclerosis (ALS), with glutamate-induced excitotoxicity believed to represent a primary mechanism of ALS pathogenesis. *In vivo* evidence of glutamate abnormality in ALS patients, however, remains inconsistent, likely reflecting heterogeneity in the severity of underlying cortical dysfunction. The current study assessed the utility of short interval intracortical inhibition (SICI), a validated marker of upper motor neuron (UMN) dysfunction in ALS, to stratify cortical motor metabolite abnormalities, as determined by proton magnetic resonance spectroscopy (^1^H-MRS). Serial ^1^H-MRS data were acquired over 2.5 years for two ALS participants with contrasting profiles of progressive motor dysfunction as a pilot study. Longitudinal monitoring of these participants demonstrated stable cortical motor metabolite concentrations in the participant with lower motor predominant disease presentation but progressive changes in glutamate–glutamine (Glx) and N-acetylaspartate (NAA) concentrations in the participant with a classical ALS presentation. Fifty-four participants (34 ALS; 20 control) were prospectively recruited for a formal study. All patients underwent threshold-tracking transcranial magnetic stimulation) and were classified as having high (>5.5%; H-SICI) or low (≤5.5%; L-SICI) cortical motor inhibition. Matching 3T single-voxel ^1^H-MRS data were acquired from the hand region of the motor cortex for all participants at baseline, with a subset of patients (*n* = 10) longitudinally assessed at 6 months. Dissociable patterns of pathological change in NAA and Glx/NAA metabolites were observed at baseline and longitudinally in ALS. At baseline, L-SICI ALS participants with increased cortical motor excitability demonstrated a significant bilateral reduction in NAA and elevated Glx/NAA metabolite concentrations (*P*-values < 0.03), contrasting to H-SICI ALS participants, where the neurochemical concentration was preserved. At follow-up, H-SICI patients demonstrated a trend towards elevated Glx and Glx/NAA in the left motor cortex (*P*-values ≤ 0.06). In contrast, L-SICI patients demonstrated stable concentrations of Glx but further reductions in NAA ratio (*P* = 0.04). Cortical excitability and brain neurochemical profile abnormalities reflect evolving states of UMN dysfunction in ALS. Elevated Glx/NAA metabolite concentration underlies greater cortical motor dysfunction in ALS. Longitudinal ^1^H-MRS holds potential prognostic utility for clinical monitoring of ALS disease trajectory.

## Teaser

Tu *et al*. report that threshold-tracking transcranial magnetic stimulation stratifies the cortical motor profile of metabolite abnormalities in patients with amyotrophic lateral sclerosis. Patients demonstrated a distinct profile of reduced N-acetylaspartate and an elevated glutamate/N-acetylaspartate ratio associated with altered cortical motor excitability.

## Graphical Abstract

**Graphical Abstract:**
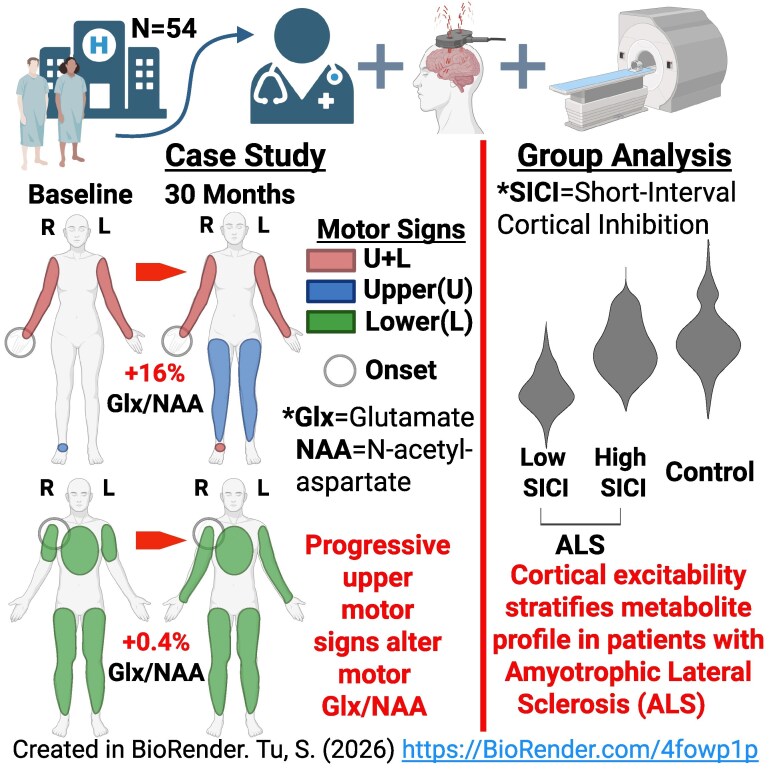

## Introduction

The evolution of cortical motor excitability, marked by a reduction or absence of short interval intracortical inhibition (SICI),^1^ is an early-stage biomarker of upper motor neuron (UMN) dysfunction in amyotrophic lateral sclerosis (ALS). Disturbances in cortical excitability have been documented across independent cohorts of sporadic and familial ALS patients,^2-6^ including the advent of abnormal SICI in asymptomatic genetic mutation carriers who subsequently developed clinical features of ALS.^7^ Reduced SICI in ALS is believed to represent an imbalance of intra-cortical inhibitory and excitatory neural circuitry, namely the degeneration of inhibitory cortical circuits, particularly inhibitory inter-neuronal function,^8-10^ accompanied by excessive excitation of high-threshold excitatory pathways.^1,11^ Extensive evidence from cultured human patient tissue and animal models of ALS indicates glutamate-associated excitotoxicity, the main excitatory neurotransmitter in the brain, is a primary mechanism underlying selective motor neuron death.^3,12^ Collectively, these studies indicate increased susceptibility and reduced capacity to modulate excess influx of intracellular calcium and sodium in ALS motor neurons,^13-16^ inducing neuronal death.

Clinically, the glutamate antagonist Riluzole^17^ is a long-standing therapeutic agent that has demonstrated a reliable disease-modifying effect for patients with ALS, with short-term and partial normalization of cortical motor excitability,^18^ associated with improved patient prognosis.^2,19^ Consequently, aberrant changes in motor-associated glutamate concentration hold a potential molecular basis for development as a biomarker of ALS pathology. To date, however, the utility for monitoring progressive pathological change in focal glutamate concentration across cortical motor regions has proven difficult in ALS.^20^

*In vivo* quantification of glutamate concentration in the motor cortex using non-invasive proton magnetic resonance spectroscopy (^1^H-MRS) has identified mixed pathological patterns in ALS.^20^ Similarly, evaluating the accuracy of MRS quantification of *in vivo* brain metabolite concentration is difficult as the ‘ground truth’ remains hidden and existing studies span low (1.5T) to high (7T) MR field strengths. The ^1^H-MRS technique benefits significantly from increased field strength in the form of increased signal-to-noise ratio and chemical shift dispersion, resulting in greater precision for resolving complex metabolite peaks.^21^ Of relevance, existing ^1^H-MRS imaging studies in ALS cohorts performed at 7T field strength^22-24^ have demonstrated variable patterns of pathological glutamate change, mirroring reports from traditional 1.5T^25-28^ and 3T^29-33^ MRI studies. This suggests that the variability in endogenous glutamate concentration in the motor cortex reflects underlying biological variability across ALS patients rather than technical limitations. As such, a primary research focus is to improve the clinical interpretation of motor-associated glutamate concentration as a reliable biomarker of disease in ALS. The application of MRS to characterize metabolic disturbances in ALS as a clinically sensitive marker of evolving cortical motor pathology,^34^ as well as therapeutic efficacy,^35,36^ is long-standing. Notably, N-acetylaspartate (NAA) has been highlighted as a relevant disease marker sensitive to the cortical motor response of Riluzole,^35,36^ as well as a surrogate marker reflecting broader structural and functional cortical motor network dysfunction^34^ in ALS.

With this background, the current study aimed to explore whether clinically validated threshold-tracking transcranial magnetic stimulation (TMS) stratification of cortical dysfunction could dissect motor-associated metabolite profiles in ALS.^1^ Specifically, whether there is a dissociation in glutamate concentration profile associated with the presence of cortical motor excitability as defined by previous large-scale validated thresholds of percentage change in SICI in ALS.^37,38^ We hypothesized that elevated glutamate in the motor cortex could serve as a consistent disease signature of ALS pathology reflecting increased severity of UMN dysfunction. Furthermore, the sensitivity of glutamate as a marker of disease in ALS is improved by normalizing for local differences in neuronal integrity, as indicated by NAA.^20^ Comparative analyses between metabolite abnormality and the sensitivity of cortical thickness changes in the cortical arm region, a clinical marker sensitive to progressive cortical degeneration in ALS,^39^ were also performed.

## Materials and methods

### Participants

Study investigations began with a longitudinal case study performed on two ALS patients (Patients A and B) with contrasting profiles of progressive upper and lower motor neuron dysfunction (UMN; LMN). Serial ^1^H-MRS was performed between 2020 and 2022, over a total duration of 2.5 years for both patients. Patient A was scanned at 5 timepoints; Patient B was scanned at 3 timepoints. Preliminary findings formed the basis to inform a longitudinal group-based study.

Thirty-four newly diagnosed non-familial ALS patients without dementia were prospectively recruited for the group study from a tertiary referral multi-disciplinary MND Clinic. A demographically well-matched cohort of 20 healthy control participants was recruited in parallel. The clinical diagnosis of ALS was made in accordance with the Gold Coast criteria.^40^ All patients underwent comprehensive neurological examination, neurophysiological assessment, and a research MRI scan on the same visit. Disease involvement and level of disability were assessed using the revised ALS functional rating scale (ALSFRS-R),^41^ a four-domain questionnaire scored between 0 and 48, with a lower score reflecting greater disability. Disease progression rate (DPR) was calculated as the difference from maximum ALSFRS-R total score, expressed as average points lost per month since disease onset. Severity of upper and lower limb muscle weakness across six muscle groups (shoulder abductors, elbow flexors, wrist extensors, hip flexors, knee extensors, foot dorsiflexors) was assessed and graded from 0 to 5 in accordance with the Medical Research Council (MRC) scale for muscle strength.^42^ Clinical UMN burden was graded between 0 and 38, totalling the number of pathological signs observed on examination (Supplementary Table 1). All patients were classified according to King’s staging of disease severity.^43^

Participant consent was obtained according to the Declaration of Helsinki. Ethical approval for this study was obtained from the University of Sydney (HREC 2021/283) and the South-Eastern Area Health Service (2021/ETH00559) ethics committees.

### MRI acquisition

All participants were scanned using a 3T GE Discovery MR750 scanner with a 32-channel head coil. A subset of 10 ALS patients completed a 6-month follow-up MRI scan. Whole-brain volumetric T1-weighted MRI scans were acquired using a magnetization-prepared rapid gradient echo sequence (TE/TR = 2.3/6.2 ms; flip angle = 12°; matrix size = 256 × 256, 204 slices, 1 mm^3^ isotropic). Consecutive single-voxel ^1^H-MRS using point-resolved spectroscopy (PRESS) acquisitions were performed (TE/TR = 35/2000 ms; 20 mm^3^ isotropic; 128 averages), with two chemical shift-selective imaging pulses for water suppression, for the left and right motor cortices, respectively. Voxels were centred on the ‘hand’ region of the motor cortex, guided by anatomical localization of the characteristic sigmoidal hook projecting posteriorly from the precentral gyrus along the central sulcus using each participant’s corresponding T1 image. Voxel placement began in the axial plane and was adjusted in the coronal and sagittal planes to exclude any dura while maximizing grey matter inclusion. Spectra were shimmed to achieve a full-width half maximum of <13 Hz. At 6-month follow-up, a voxel mask of each patient’s baseline ^1^H-MRS acquisition was used to ensure accuracy of voxel placement.

### Proton magnetic resonance spectroscopy

^1^H-MRS data were post-processed using LCModel.^44^ To minimize subject-specific variation, metabolite concentrations were considered as a relative ratio to total creatine (Cr), derived from the sum of Cr and phosphocreatine. Cr and phosphocreatine are present in all major cell types and play a crucial role in maintaining energy homeostasis. Consequently, Cr is widely used as an internal reference to normalize against other metabolite signals. Spectra were quantified using a standard PRESS TE = 35 ms basis set of 15 metabolites, incorporating macromolecule and baseline fitting. Qualitative visual inspection of all spectra was performed by two independent raters, blinded to participant information, to ensure consistent fitting of metabolite peaks. Consensus was reached to exclude three spectral datasets (ALS = 1; control = 2) due to poor quality of metabolite peak fit. The reliability of metabolite quantification was characterized by modelling the normal distribution of the estimated percentage standard deviation on a per-metabolite basis. Only metabolites demonstrating Cramer–Rao lower bounds ≤15% were included for further analysis. In accordance with *a priori* hypotheses of altered cortical motor excitability,^2,3^ comparative planned analyses of metabolite concentration were restricted to pooled glutamate and glutamine (Glx) and NAA. Additionally, baseline ^1^H-MRS data of the right motor cortex were not acquired for 2 ALS participants due to discomfort maintaining a supine position without supporting head elevation, resulting in a shorter scan.

### Assessment of cortical motor function

Threshold-tracking TMS was performed as part of comprehensive clinical and neurophysiological assessments at the Sydney Forefront MND Clinic, using validated protocols with MagXite software,^45,46^ as per the Sydney protocol.^47^ Cortical function of the motor hemisphere contralateral to the dominant hand was assessed in all patients using paired-pulse threshold-tracking TMS to quantify per cent change in SICI as a robust measure of cortical excitability.^1^ Briefly, TMS examinations were performed using two Magstim 200 stimulators connected in BiStim mode and a 90 mm circular coil. Participants were seated comfortably in a purpose-built chair, with arms resting on a pillow and forearms midway between pronation and supination. The coil, initially centred over the vertex, was adjusted in the antero-posterior and medial-lateral directions and positioned at which a consistent motor evoked potential (MEP) response (>50 μV) was attained from the abductor pollicis brevis. Coil position was fixed using a stand. Resting motor threshold (RMT) was then determined, defined as the stimulus intensity required to produce and maintain a peak-to-peak MEP response of 0.2 mV (±20%). Subthreshold conditioning stimulus was set to 70% of RMT and test intensity was varied to target a fixed MEP of 0.2 mV (±20%). SICI was recorded in a serial ascending manner over interstimulus intervals (ISI): 1, 1.5, 2, 2.5, 3, 3.5, 4, 5 and 7 ms. Live visual and auditory feedback was provided to the participant and monitored by the assessor throughout recording to exclude activation of the target muscle. Non-stimulus-related muscle activation (i.e. fasciculations) during the SICI protocol resulted in an immediate pause in stimulus delivery and recording. All recordings were visually examined by the assessor for any notable change in RMT, impacting quantification of per cent change of all subsequent SICI intervals, and the protocol was repeated as necessary. Detailed methodology of the paired-pulse threshold-tracking TMS protocol, and validation of SICI in ALS, has been extensively reported.^1,3,37,38^ All ALS patients with a per cent change in SICI ≤5.5 were categorized in the low SICI (L-SICI) group, with remaining patients considered to have a high SICI (H-SICI).^37,38^ TMS stratification was performed as an objective marker to identify ALS subtypes (i.e. L-SICI) with a relatively predominant UMN profile of disease pathology. To our knowledge, no historical healthy control assessed using the current TMS protocol fell below the threshold of 5.5% change in SICI.

### Cortical thickness

Each individual’s structural MRI scan was processed using the FreeSurfer V.6.0 software package (https://surfer.nmr.mgh.harvard.edu). T1-weighted images were first processed through the standard FreeSurfer recon-all pipeline, correcting for motion and intensity inhomogeneity, removal of non-brain tissue and segmentation of white and grey matter. Brodmann labels of the primary motor cortex (BA4) were used to calculate average cortical thickness of the arm region by dividing each hemisphere into 30 equidistant segments (leg = 1–8; arm = 9–19; bulbar = 20–30).^39^ Segments were sequentially numbered from medial to lateral, and cortical thickness measurements were corrected for total intracranial volume.

### Statistical analyses

Statistical analyses were carried out using SPSS V.28.0. The Shapiro–Wilk test was used to test normality. Clinical features were assessed using analysis of variance (ANOVA) across patients and control cohorts, or Kruskal–Wallis test where appropriate. Group differences in baseline metabolite concentrations and cortical thickness were assessed using analysis of covariance (ANCOVA) across patient and control cohorts. Longitudinal change in metabolite concentration was analysed using repeated measures ANCOVA. Partial correlation was performed to examine the relationship between metabolite concentration and clinical measures of disease severity and progression rate. Baseline group comparisons and clinical correlations were adjusted for multiple comparisons using Bonferroni correction, separately for each brain hemisphere, based on the number of metabolites of interest. Bonferroni-adjusted *P*-values were reported. Age and gender were controlled for as covariates across all comparative group analyses and clinical correlations.^48^ Receiver operating characteristic (ROC) curves of sensitivity and specificity were used to assess the diagnostic accuracy of metabolite concentration for modelling disease from controls. Area under the curve (AUC) and likelihood ratio (LR) were calculated.

### Case example—longitudinal ^1^H-MRS monitoring

**Figure 1:**
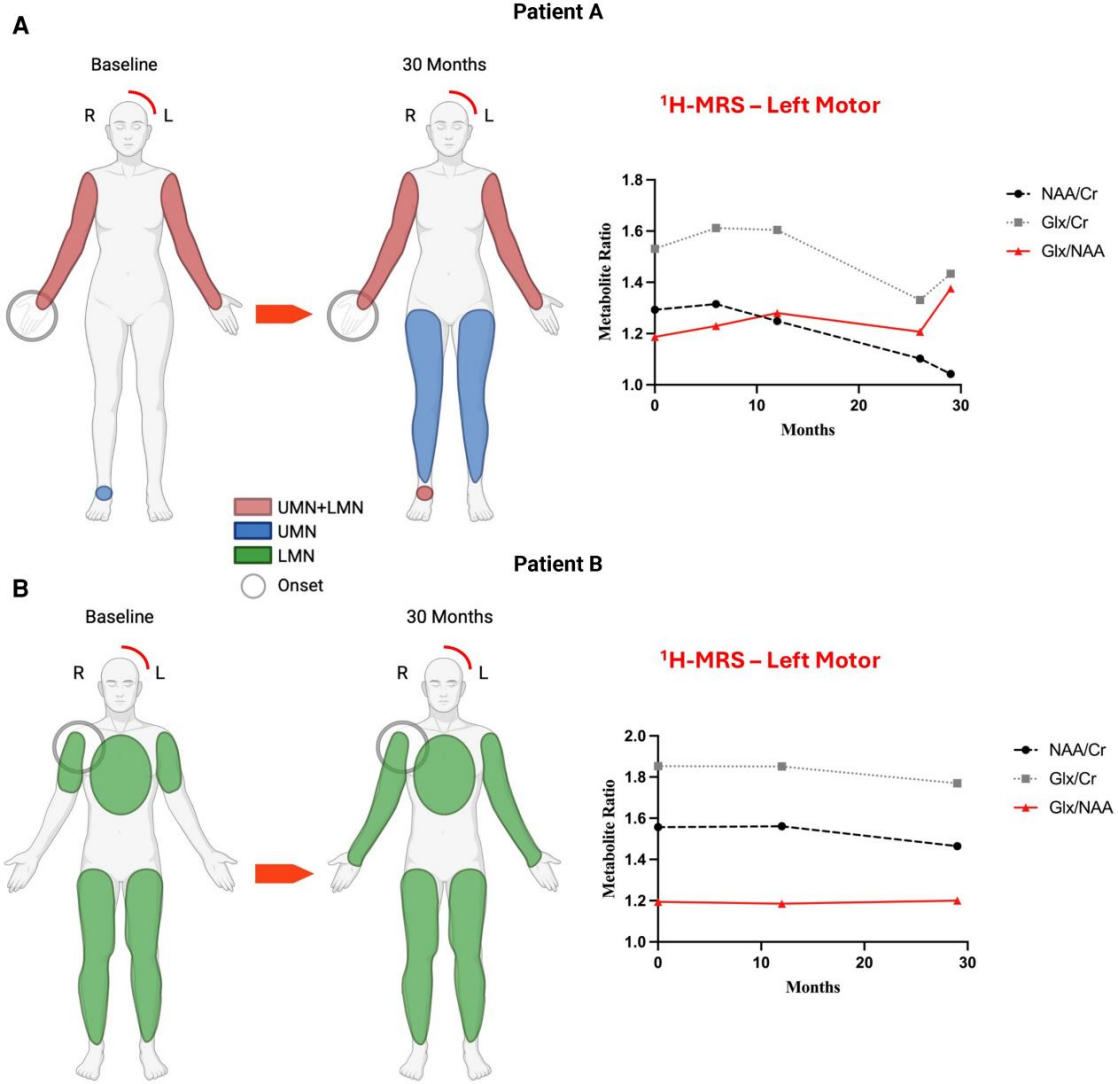
**Longitudinal case example.** Comparison of longitudinal cortical motor metabolite concentration alterations over 30 months in two ALS patients with contrasting profiles of progressive upper and lower motor neuron dysfunction. [Patient A] Presented with primary upper and lower motor neuron (UMN; LMN) clinical features in the upper limbs. Progression was characterized by development of UMN features in the lower limbs (**A**). ^1^H-MRS indicated dynamic metabolite alterations with a notable increase in Glx/NAA. [Patient B] Presented with LMN-predominant clinical features across proximal upper limbs and lower limbs. Progression was characterized by distal spread of LMN features involving both upper limbs (**B**). ^1^H-MRS indicated a stable longitudinal profile of cortical motor metabolite concentration. Figure created in BioRender. Tu, S. (2026); https://BioRender.com/85q35a9. *Note: proton magnetic resonance spectroscopy (^1^H-MRS); N-acetylaspartate (NAA); glutamate–glutamine (Glx); creatine (Cr).

Serial ^1^H-MRS was performed over 2.5 years in two ALS participants with contrasting profiles of progressive UMN and LMN dysfunction on neurological examination, and a relatively slow rate of disease progression suitable for long-term follow-up MRI (Fig. 1). Threshold-tracking TMS indicated both patients had ≤5.5% SICI at initial clinical examination in the dominant motor hemisphere. Longitudinal ^1^H-MRS profiles demonstrated a dissociation between Patients A and B, consistent with the evolution of UMN and LMN clinical features, respectively. Clinical progression in Patient A was characterized by a notable development of hyperreflexia in both lower limbs and a 16% increase in Glx/NAA ratio between baseline and final time-point. In contrast, clinical progression in Patient B was characterized by the distal spread of muscle wasting in both upper limbs, a persistence of an LMN-predominant phenotype. Cortical motor metabolite profiles remained stable over the follow-up period, with a 0.4% increase in Glx/NAA between baseline and final time-point.

#### Patient A

A 47-year-old woman presented with a 2-year history of minimally progressive weakness of her dominant right hand. Examination revealed isolated mild pyramidal weakness in the right hand accompanied by fasciculations in both upper limbs. Brisk reflexes were present across both upper limbs and right ankle, and a positive right Hoffman’s sign. Clinical progression was evidenced between first and final MRI scans. At the final timepoint, examination revealed significant wasting and weakness of the right upper limb, progressive spreading of weakness developing in the left upper limb and development of mild right ankle dorsiflexion weakness. Brisk reflexes were present across both upper limbs with evidence of significant spread across lower limbs. Disease progression was relatively slow, as reflected by a reduction in ALSFRS-R total score of 7 points (45–38) over 30 months, following initial diagnosis.

#### Patient B

A 69-year-old gentleman presented with a 3-year history of upper limb weakness in the right shoulder, progressing to the left proximal upper limb. Examination revealed prominent bilateral muscle wasting of the proximal upper limbs accompanied by fasciculations in both proximal upper limbs, chest and lower limbs. Muscle bulk appeared preserved in distal hand muscles, with very mild weakness observed for right wrist and finger extension. No muscle wasting or weakness was observed across lower limbs. Reflexes were generally depressed across both upper and lower limbs. Minimal clinical progression was evidenced between first and final MRI scans. At the final timepoint, examination revealed bilateral mild progressive wasting of the proximal limbs, spreading distally to affect intrinsic hand muscles typical of the ‘split-hand’ presentation seen in ALS.^2^ No muscle wasting or weakness was observed across lower limbs. Disease progression was relatively slow, as reflected by a reduction in ALSFRS-R total score of 3 points (47–44) over 30 months, following initial diagnosis.

## Results

### Participant data and demographics

In terms of clinical presentations (Supplementary Table 2), the majority of ALS patients in the group study presented with limb disease onset (74%), while the remainder demonstrated bulbar disease onset (26%). Moderate level of disability and rate of disease progression were evident in ALS as indicated by a mean ALSFRS-R total score of 38.5 (SD = 6.8) and DPR of 0.82 (SD = 1.2) at initial diagnosis. Control participants (*n* = 20) were prospectively recruited in parallel to ALS patients (*n* = 34) to ensure cohorts were well-matched across demographic variables, including age, years of education, gender and handedness (*P*-values > 0.1).

TMS stratification of ALS patients based on per cent change in SICI identified 59% of ALS patients as exhibiting increased cortical excitability (L-SICI; *n* = 20), confirming a significant level of cortical motor dysfunction (Table 1). With respect to site of initial symptom onset, the L-SICI cohort comprised 46% of total patients with an upper limb presentation, 75% of lower limb presentation and 55% of bulbar presentation. With respect to King’s staging of disease severity, the L-SICI cohort comprised 67% of patients classified as Stage 1, 45% of patients in Stage 2, 86% of patients in Stage 3 and 25% of patients in Stage 4. The L-SICI cohort demonstrated greater clinical UMN features at examination (*P* = 0.02) but appeared otherwise identical to the H-SICI cohort in terms of disease severity, duration and site of onset (*P*-values > 0.16). No statistically significant distinction in DPR was observed between TMS-stratified ALS cohorts (*P* = 0.41).

**Table 1:** Mean and standard deviation of participant demographics and ALS patient clinical features

	Low SICI (*n* = 20)	High SICI (*n* = 14)	Control (*n* = 20)	*P*-value
Age (y.o)	58.5 (12.2)	61.2 (8.6)	63.4 (10.2)	0.34
Education (years)	13 (2.8)	12.8 (3.7)	15.2 (4.5)	0.24
Gender (M/F)	8 M, 12 F	10 M, 4 F	9 M, 11 F	0.17
Handedness (R/L/B)	19 R, 1 L	13 R, 1 L	18 R, 1 L, 1B	0.77
Disease duration (months)	14.5 (6.9)	16.3 (8)	-	0.93
ALSFRS-R (/48)	39.4 (4.3)	39.9 (5)	-	0.32
Disease progression rate (48-ALSFRS-R/duration)	1.04 (1.8)	0.56 (0.4)	-	0.41
Site of onset (UL/LL/bulbar)	6 UL, 9 LL, 5 bulbar	7 UL, 3 LL, 4 bulbar	-	0.33
UMN score (/38)	12.8 (8.8)	6.2 (6.2)	-	**0**.02*
Riluzole (Y/N)	16 Y, 4 N	10 Y, 4 N		0.43
MRC scale				
Right (/30)	27.1 (3)	25.9 (5.2)	-	0.93
Left (/30)	26.9 (2.3)	26 (5.7)	-	0.58
Total (/60)	54 (3.7)	54.4 (5.8)	-	0.52
King’s staging				0.17
1	8 (40%)	4 (28.5%)	-	
2	5 (25%)	6 (43%)	-	
3	6 (30%)	1 (7%)	-	
4	1 (5%)	3 (21.5%)	-	

ALS patients were stratified based on severity of upper motor neuron dysfunction as defined by per cent change in short interval intracortical inhibition (SICI).

^*^Significance at *P* < 0.05. Revised ALS Functional Rating Scale (ALSFRS-R); upper motor neuron (UMN); upper limb (UL); Medical Research Council (MRC).

### ^1^H-MRS analyses

**Figure 2:**
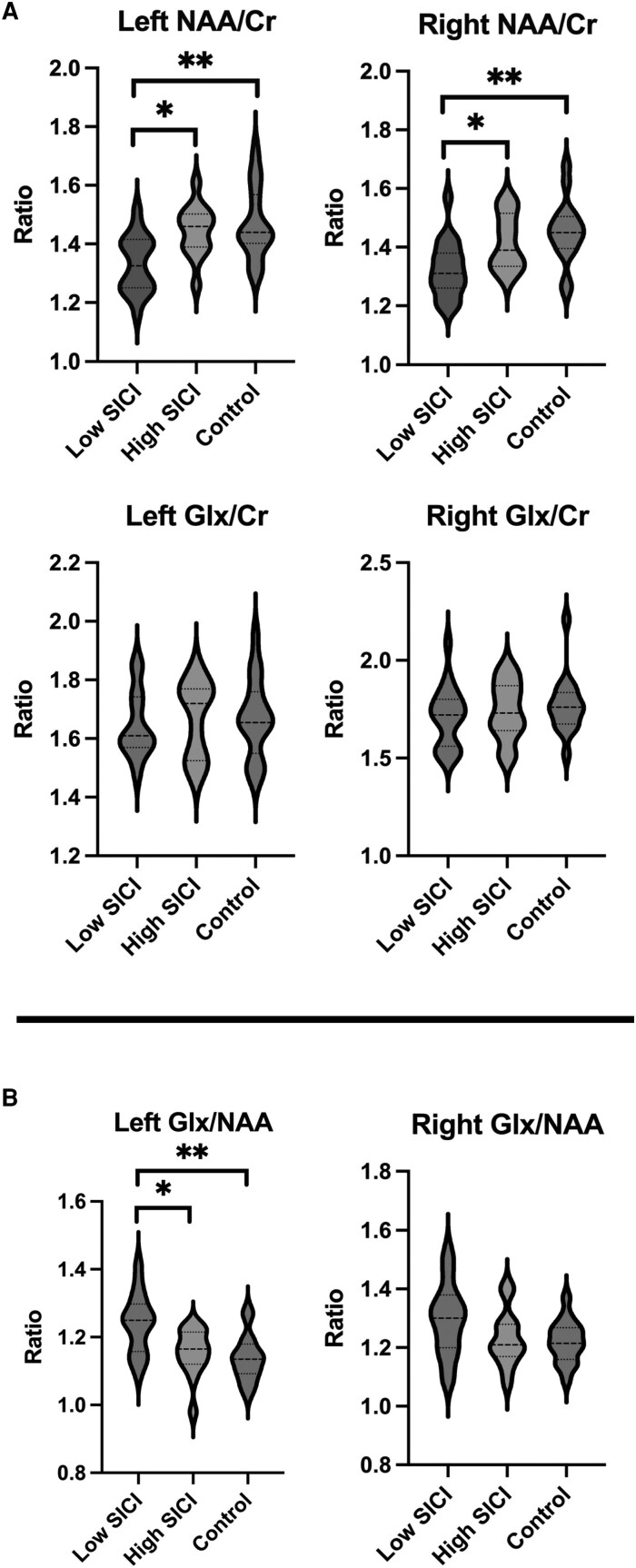
**Cortical motor metabolite profile.** Metabolite concentrations (**A**: NAA/Cr, Glx/Cr; **B**: Glx/NAA) in the hand region of the motor cortex in 34 ALS patients and 20 healthy control participants. ALS patients were stratified based on severity of upper motor neuron dysfunction as defined by per cent change in short interval intracortical inhibition (SICI). Median and interquartile range are shown. Group differences were assessed using ANCOVA, covarying for age and gender. * Significance at *P* < 0.05; ** *P* < 0.01. Note: ^1^H-MRS of the right hemisphere was not acquired for one ALS patient in the high (H-SICI) and low (L-SICI) SICI cohorts, respectively; N-acetylaspartate (NAA); glutamate–glutamine (Glx); creatine (Cr).

Metabolite concentrations of NAA and Glx were quantified in the hand region of the left and right motor cortices of all ALS and control participants. L-SICI demonstrated significantly altered metabolite profiles relative to H-SICI and control cohorts (Fig. 2). A consistent bilateral reduction was observed for NAA/Cr (left: mean = 1.33, SE = 0.02, *P*-values < 0.02; right: mean = 1.32, SE = 0.03, *P*-values < 0.05), and elevated left Glx/NAA ratio (mean = 1.24, SE = 0.02, *P*-values < 0.02) in L-SICI ALS patients. No significant metabolite alterations were observed between H-SICI and control cohorts (*P*-values > 0.31). The same pattern of comparative group differences was observed after excluding the 8 ALS patients not currently receiving Riluzole at baseline assessment, with the exception of left NAA/Cr, whereby a significant difference between the L-SICI and H-SICI cohorts was no longer observed (*P* = 0.16; Supplementary Fig. 1).

The same pattern of altered metabolite profiles was observed between the combined ALS cohort and control cohort (Supplementary Fig. 2). A consistent bilateral reduction was observed for NAA/Cr (left: mean = 1.38, SE = 0.02, *P* < 0.01; right: mean = 1.36, SE = 0.02, *P* < 0.01), and elevated left Glx/NAA (mean = 1.2, SE = 0.01, *P* = 0.03) in ALS patients. Correlations for each SICI ISI (1–7 ms) with NAA and Glx metabolite concentrations are provided in Supplementary Table 3.

### Longitudinal ^1^H-MRS analyses

A clinically (ALSFRS-R; disease duration) matched subset of ALS patients (L-SICI = 5; H-SICI = 5) was longitudinally scanned at baseline and 6 months. L-SICI and H-SICI ALS cohorts demonstrated distinct longitudinal metabolite profiles of change over the initial 6 months following clinical diagnosis (Table 2). Notably, L-SICI demonstrated a significant reduction in left NAA/Cr (*P* = 0.04), but stable Glx/Cr (*P* > 0.95) and Glx/NAA (*P*-values > 0.14) concentrations. In contrast, H-SICI demonstrated a trend towards elevated left Glx/Cr (*P* = 0.05) and Glx/NAA (*P* = 0.06), but stable NAA/Cr (*P*-values > 0.47) concentration. No significant group differences were observed between baseline metabolite profiles of the longitudinal subset of L-SICI and H-SICI ALS patients (*P*-values > 0.06).

**Table 2:** Mean and standard error of metabolite concentrations in the hand region of the motor cortex in a subset of 10 ALS patients longitudinally assessed at baseline and 6-month follow-up

	Metabolite	Baseline	6-Month	*P*-value
**Low SICI**				
**(*n*** **=** **5)**	NAA/Cr			
	Left	1.36 (0.04)	1.31 (0.03)	**0**.04*
	Right	1.29 (0.05)	1.25 (0.03)	0.26
	Glx/Cr			
	Left	1.6 (0.04)	1.61 (0.03)	0.96
	Right	1.69 (0.09)	1.69 (0.05)	0.96
	Glx/NAA			
	Left	1.18 (0.04)	1.23 (0.04)	0.34
	Right	1.31 (0.03)	1.35 (0.03)	0.43
**High SICI**				
**(*n*** **=** **5)**	NAA/Cr			
	Left	1.42 (0.02)	1.38 (0.06)	0.48
	Right	1.38 (0.03)	1.4 (0.06)	0.6
	Glx/Cr			
	Left	1.6 (0.06)	1.69 (0.08)	0.05
	Right	1.75 (0.03)	1.73 (0.03)	0.58
	Glx/NAA			
	Left	1.13 (0.06)	1.22 (0.07)	0.06
	Right	1.26 (0.04)	1.23 (0.08)	0.58

Patients were stratified based on severity of upper motor neuron dysfunction as defined by per cent change in short interval intracortical inhibition (SICI).

^*^Significance at *P* < 0.05, uncorrected for multiple comparisons.

The combined ALS cohort demonstrated a significant longitudinal reduction in left NAA/Cr (*P* = 0.03) and elevated left Glx/NAA (*P* = 0.02) (Supplementary Table 4).

### Clinical correlations

**Figure 3:**
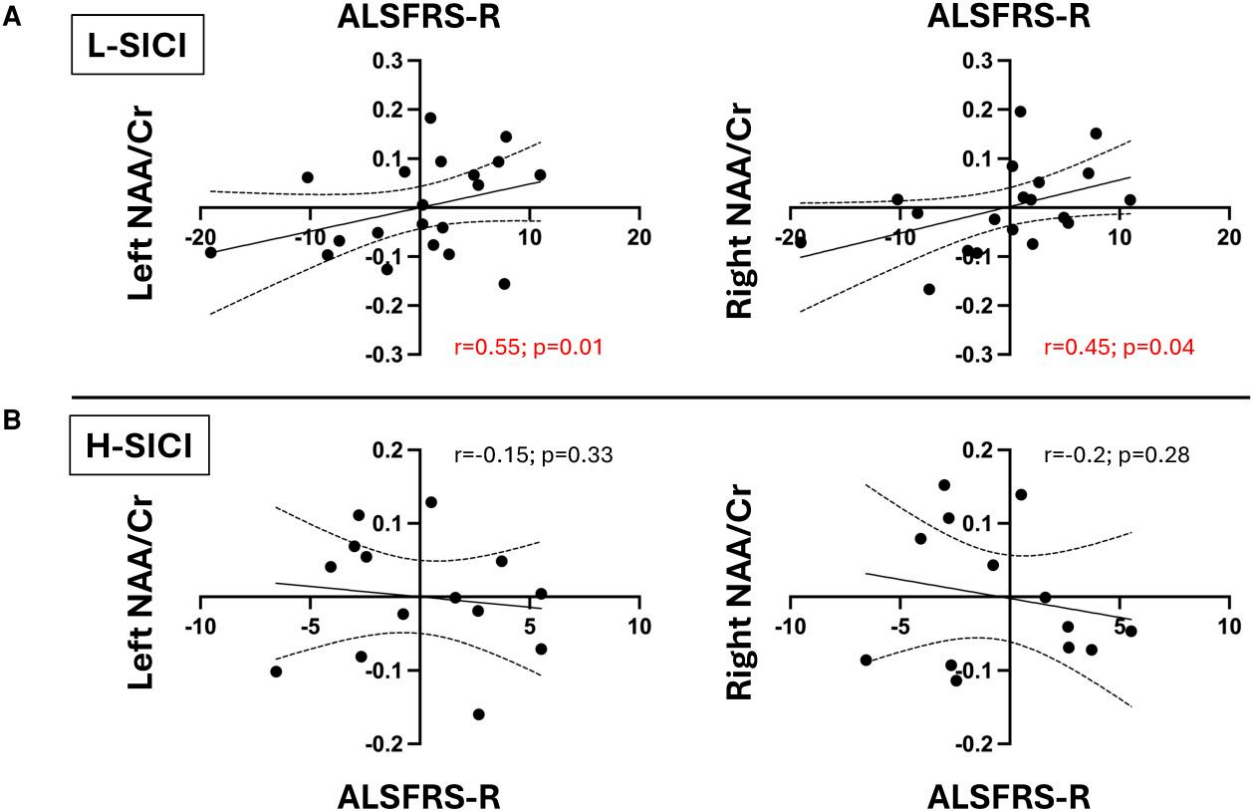
**Metabolite clinical correlations.** Clinical correlation between NAA/Cr metabolite concentration and ALSFRS-R total score in the low short interval intracortical inhibition (L-SICI; *n* = 20; **A**) and high short interval intracortical inhibition (H-SICI; *n* = 14; **B**) ALS patient cohorts. Partial correlations were performed; residuals are shown controlling for age and gender. Data points represent the measurement from individual participants. Note: ^1^H-MRS of the right hemisphere was not acquired for one ALS patient in the H-SICI and L-SICI cohorts, respectively; N-acetylaspartate (NAA); creatine (Cr).

Planned clinical correlations were performed between NAA and Glx metabolites of interest to examine relationships with disease severity and progression (Fig. 3). A significant positive correlation was observed between increased bilateral NAA/Cr metabolite concentration and reduced functional impairment on the ALSFRS-R total score (left: R = 0.55, *P* = 0.01; right: R = 0.45, *P* = 0.04) in the L-SICI cohort. No significant relationship was observed for NAA/Cr in the H-SICI cohort. No significant relationship was observed for Glx/Cr and Glx/NAA metabolites (*P*-values > 0.08).

### Cortical thickness

Average cortical thickness was examined for the arm region of the primary motor cortex (BA4) across ALS patient cohorts and healthy controls (Supplementary Table 5). No significant group differences were observed (*P*-values > 0.45).

### ROC analyses

**Figure 4:**
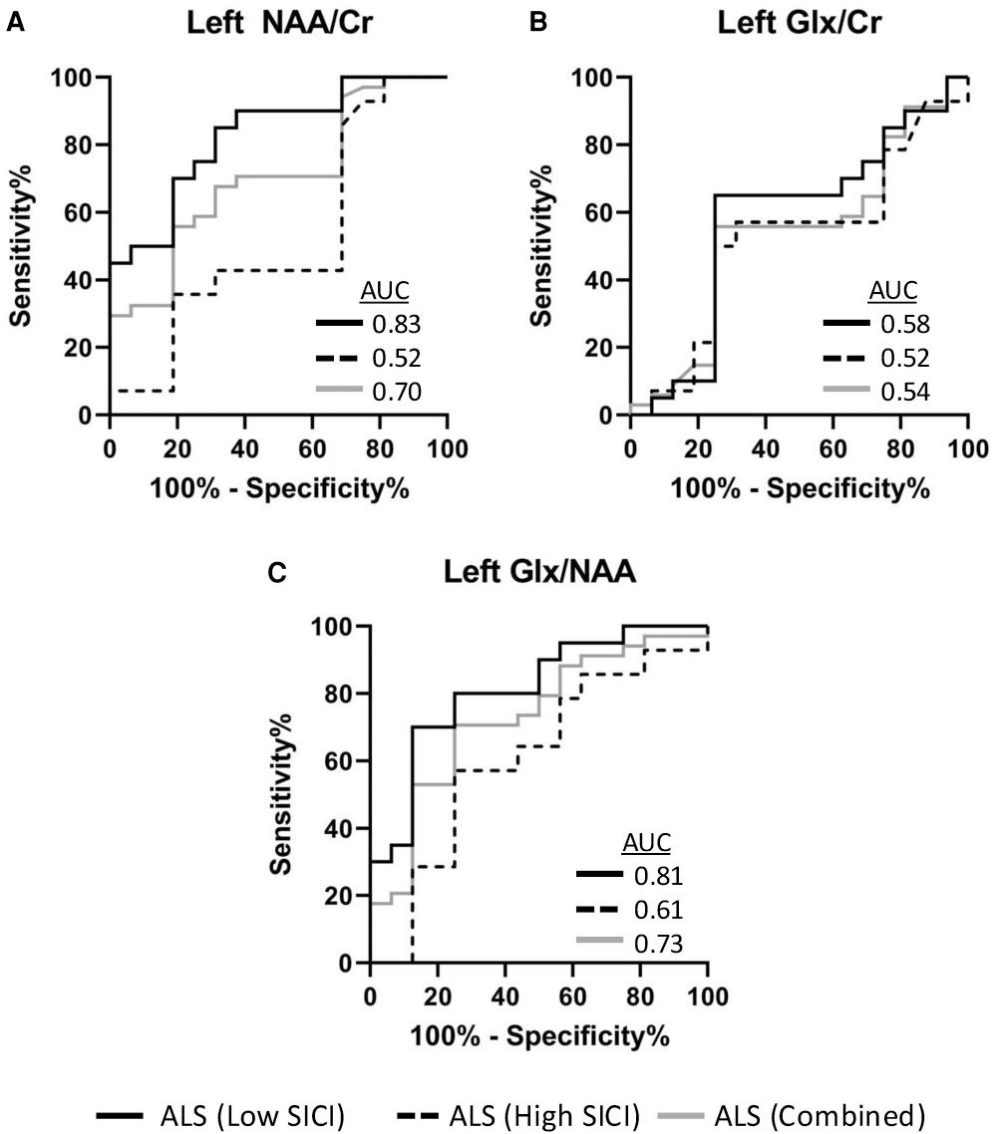
**Metabolite ROC curves.** ROC curves of NAA (**A**), Glx (**B**) and Glx/NAA (**C**) metabolite concentrations in the hand region of the left motor cortex. The sensitivity and specificity of metabolite change were calculated between the ALS cohort (low SICI, *n* = 20; high SICI, *n* = 14) and healthy controls (*n* = 20). Patients were stratified based on severity of UMN dysfunction as defined by per cent change in short interval intracortical inhibition (SICI). Area under the curve (AUC) is reported. *Note: N-acetylaspartate (NAA); glutamate–glutamine (Glx); creatine (Cr).

ROC curves were calculated for NAA and Glx metabolite concentrations in the left motor cortex as predictors for differentiating ALS from healthy controls (Fig. 4). AUC values were highest in the low SICI ALS cohort for both NAA (AUC = 0.83, SE = 0.07, 95% CI = 0.7–0.96, *P* < 0.01) and Glx/NAA (AUC = 0.81, SE = 0.07, 95% CI = 0.67–0.95, *P* < 0.01). AUC of the ALS cohort as a whole was also able to significantly model disease from controls based on NAA (AUC = 0.7, SE = 0.08, 95% CI = 0.55–0.85, *P* = 0.02) and Glx/NAA (AUC = 0.73, SE = 0.08, 95% CI = 0.58–0.88, *P* < 0.01). The high SICI ALS cohort, as well as standalone Glx concentration, was a poor predictor that could not model disease from controls (*P* values > 0.32).

The sensitivity and specificity of NAA and Glx/NAA metabolite change were assessed (Supplementary Table 6). Optimal disease thresholds included a reduced NAA of <1.4 (L-SICI: sensitivity = 75%, specificity = 75%, LR = 3; ALS: sensitivity = 59%, specificity = 75%, LR = 2.4), and elevated Glx/NAA of >1.15 (L-SICI: sensitivity = 80%, specificity = 75%, LR = 3.2; ALS: sensitivity = 71%, specificity = 75%, LR = 2.8).

## Discussion

The present study demonstrated that altered cortical excitability stratifies metabolite abnormalities in sporadic ALS and highlights a potential complementary approach for patient phenotyping. ^1^H-MRS of the motor cortex indicated a significant reduction in neuronal integrity (NAA/Cr) and elevation of neuronal excitation (Glx/NAA) in ALS with reduced SICI. Furthermore, longitudinal analyses indicated a notable dissociation in Glx and NAA profiles between SICI (%) stratified ALS cohorts. Importantly, cortical motor metabolite abnormalities appear to precede measurable disease-related changes in cortical thickness, a structural MRI measure of neuronal loss. The patient case example demonstrated qualitative long-term changes in cortical motor metabolite profiles accompany spread of UMN clinical presentation following initial diagnosis. Current findings emphasize the nature of evolving cortical motor dysfunction in ALS and the need for complementary biomarkers to model inherent disease heterogeneity for clinical monitoring and stratification into prospective therapeutic trials.

### Cortical inhibition and glutamate-associated motor pathophysiology in ALS

Reduced inhibitory corticomotoneuronal function represents a robust neurophysiological signature of ALS, as measured by threshold-tracking TMS.^1,38^ As such, much of the TMS literature has focused on the molecular bases of GABAergic-mediated inhibitory inter-neuronal dysfunction driving a ‘dying-forward’ mechanism of altered cortical excitability.^8^ However, the metabolite signature of ALS, as measured by non-invasive ^1^H-MRS imaging, has demonstrated limited evidence of significant abnormalities in GABA metabolite concentration in ALS patients.^31,49^ More recent independent studies at both 3T and 7T MR field strength indicated no significant change in cortical motor GABA concentration in ALS.^24,33^ Both studies instead indicated abnormally elevated concentration of cortical motor glutamate in ALS, as well as reduced NAA. Our current findings highlight the potential role of TMS-quantified inter-neuronal dysfunction for interpreting the variability of glutamate and NAA abnormalities in ALS. Specifically, in patients whose averaged SICI (%) has not fallen below the expected threshold typical for ALS,^37,38^ metabolite abnormalities do not significantly differ relative to matched healthy controls.

Our longitudinal comparison over the first 6 months following an initial diagnosis indicated a trend towards increased Glx concentration in patients with a high SICI, remaining stable in patients with a low SICI, at baseline. This could help to explain the limited benefits of existing glutamatergic approaches to ALS therapies,^50,51^ targeting a consequence rather than driver of cortical motor pathology in ALS. Quantitative changes in cortical motor glutamate abnormalities are detectable via clinically feasible MRI sequences in ALS and hold potential for clinical phenotyping and monitoring purposes. Complementary TMS and MRS assessment is likely to result in more meaningful and robust interpretation of excitatory and inhibitory processes, sensitive to cortical motor dysfunction in ALS.

### Cortical motor dysfunction and clinical presentation in ALS

A feature of the current ALS cohort is that all patients were assessed at the time of their initial diagnosis, allowing the capture of a comparative cohort with high SICI.^4^ Relative to the L-SICI cohort, ALS patients with a high SICI demonstrated less clinical UMN signs but overall did not noticeably differ in terms of demographics, disease severity or progression rate. A notable dissociation was observed whereby reduced NAA was significantly associated with more severe functional motor impairments in patients with reduced SICI. The biological basis of this dissociation is difficult to prove without further longitudinal data, as TMS assessment was only performed as part of routine clinical examination for diagnostic purposes, not at 6-month follow-up.

One hypothesis is that prior to exceeding a threshold of inhibitory dysfunction, cortical motor metabolite profiles remain in a state of heightened flux, actively modulated by changing energy demands for the regulation of intracellular Ca^2+^ homeostasis for neurotransmission.^52^ This likely has a direct impact on NAA, which is synthesized exclusively in mitochondria of neurons, acting as a surrogate marker of neuronal integrity in neurodegenerative disorders.^53^ The transition to a chronic state of altered cortical excitability, as a robust trajectory of evolving cortical dysfunction in ALS,^4^ results in a more homogeneous but elevated level of excitatory/inhibitory neuronal imbalance. As a consequence, cortical motor NAA concentration stabilizes, with further reduced levels demonstrating a meaningful association with increased severity of functional motor impairments in patients. Evidence from our earlier qualitative case-study, monitoring Glx and NAA change over 30 months, presents further evidence to support that (i) metabolite profiles remain stable in ALS with altered cortical excitability, but (ii) undergo further dynamic change with progressive spread and/or worsening of UMN symptoms.

There are a number of notable strengths implemented in the current study design. The present study has promoted direct examination of the effects of corticomotoneuronal dysfunction on *in vivo* neuronal motor metabolite profiles in ALS. Secondly, the scope of the study is hypothesis-driven, with analyses focused on advancing established knowledge of glutamate and NAA metabolite abnormalities in ALS for the purposes of improving clinical translation. A general caveat for reconciling the relative absence of GABA abnormality from the ALS neuroimaging literature, and prominence of GABAergic mediated dysfunction from the TMS field, is that TMS induces motor excitation as a targeted measure of transsynaptic inter-neuronal function while ^1^H-MRS measures relative concentration of neurochemicals based on known magnetic properties tied to their composition within a pre-defined region of interest. With respect to glutamate and GABA, ^1^H-MRS does not provide any specificity to their cellular or synaptic localization. The disconnect between TMS excitability measures of GABAergic motor function and ^1^H-MRS GABA in healthy individuals has been clearly demonstrated.^54^ There remains significant scope for further investigation to facilitate the translation of ^1^H-MRS for clinical adoption in ALS. An outstanding question is longitudinal profiles of glutamate and GABA, as well as Glx, and their association with clinical phenotypes and the phenomena of cortical excitability in ALS remains unknown. More recently, the focus has also shifted to the phenomena of cortical in-excitability, namely the inability to elicit an MEP with TMS in the absence of muscle wasting.^55^ Targeted investigations of the inter-relationships between TMS parameters, ideally emphasizing contrasting inhibitory and facilitatory protocols, with cortical motor metabolite profiles in ALS could improve current understanding of underlying mechanisms of cortical motor dysfunction as a consequence of evolving disease state. Additionally, potential underlying dissociations as a result of difference in clinical subtyping and disease staging. The primary limitation of the current study is the relatively limited longitudinal ALS sample size. A multi-centre approach through existing ALS neuroimaging consortia (i.e. NiSALS, CALSNIC) presents the ideal platform to deliver more appropriately powered longitudinal investigations.^56,57^

Our findings highlight the utility of TMS stratification and ^1^H-MRS as a complementary approach for assessing underlying cortical motor dysfunction in ALS. Longitudinal NAA and glutamate metabolite alterations reflect evolving disease presentation in ALS, demonstrating a potential role for clinical monitoring and screening for therapeutic trials. Despite the logistical challenges for implementing such techniques in a clinical setting, it is clear that no single disease marker is sufficient to capture the multi-faceted nature of ALS pathology. In a broader sense, the combination of TMS and MRI markers presents significant potential to overcome individual technical limitations of each modality for diagnostic and monitoring purposes in ALS and to untangle the dynamics between cortical motor hyper-excitability, metabolic dysregulation and disrupted neural network connectivity.

## Supplementary Material

fcag292_Supplementary_Data

## Data Availability

The datasets generated during and/or analysed during the current study are not publicly available due to sensitive patient information but are available from the corresponding author on reasonable request.
