# Supplementary material for: Cortical excitability stratifies neurochemical profiles in amyotrophic lateral sclerosis
Source: Brain Commun. 2026 Jul 28;8(4):fcag292. doi: 10.1093/braincomms/fcag292 (PMC13464032; doi:10.1093/braincomms/fcag292)
Supplement: Supplementary file 1 [file fcag292_supplementary_data.docx]

**Supplementary Table 1.** Upper motor neuron (UMN) score based on patient clinical examination.

| **Clinical Feature** | **Score** |
| --- | --- |
| Pseudobulbar Affect | /1 |
| Facial Jerk | /1 |
| Jaw Jerk | /1 |
| Pout Reflex | /1 |
| Hoffman’s Sign (L/R) | /2 |
|  |  |
| Muscle Tone |  |
| Biceps (L/R) | /4 |
| Quadriceps (L/R) | /4 |
| Ankle Clonus (L/R) | /2 |
|  |  |
| Reflexes |  |
| Bicep (L/R) | /4 |
| Tricep (L/R) | /4 |
| Supinator (L/R) | /4 |
| Finger (L/R) | /2 |
| Patellar (L/R) | /4 |
| Achilles (L/R) | /4 |
|  |  |
| **Total** | **/38** |

*L/R indicates combined total score of left and right hemispheres.

**Supplementary Table 2.** Mean participant demographics and ALS patient clinical features.

|  | **ALS**  **(n=34)** | **Control**  **(n=20)** | **P-value** |
| --- | --- | --- | --- |
| **Age (y.o)** | 59.5 (10.4) | 63.4 (10.2) | 0.27 |
| **Education (years)** | 13.2 (3.5) | 15.2 (4.5) | 0.11 |
| **Gender (M/F)** | 18 M, 16 F | 9 M, 11 F | 0.57 |
| **Handedness (R/L/B)** | 32 R, 2 L | 18 R, 1 L, 1B | 0.42 |
| **Disease Duration (months)** | 17 (11) | - | - |
| **ALSFRS-R (/48)** | 38.5 (6.8) | - | - |
| **Disease Progression Rate** | 0.82 (1.2) |  |  |
| **Site of Onset (UL/LL/Bulbar)** | 13 UL, 12 LL,  9 Bulbar | - | - |
| **UMN Score (/38)** | 9.7 (7.9) | - | - |
| **Riluzole (Y/N)** | 26 Y, 8 N |  |  |
| **MRC Scale**  **Right (/30)**  **Left (/30)**  **Total (/60)** | 26.2 (4.1)  26.4 (4)  53.5 (5.2) | -  -  - | -  -  - |
| **Kings Staging**  **1** | 12 (35%) |  |  |
| **2** | 11 (32%) |  |  |
| **3** | 7 (21%) |  |  |
| **4** | 4 (12%) |  |  |

*Revised ALS Functional Rating Scale (ALSFRS-R); Upper Motor Neuron (UMN); Upper Limb (UL); Medical Research Council (MRC)

**Supplementary Table 3.** Association between each short-interval cortical inhibition (SICI) inter-stimulus interval (ISI; 1-7ms) with NAA and Glx metabolite concentrations in the hand region of the left and right motor cortices in ALS patients. Partial correlations were performed, controlling for age and gender. Mean and standard error of each ISI are reported. R-value of the correlation between ISI and metabolite concentrations are reported. *Indicates significance at p<0.05, **indicates significance at p<0.01, uncorrected for multiple comparisons.

| **Inter-stimulus Interval (ms)** | Mean (SE) | NAA/Cr | | Glx/Cr | | Glx/NAA | |
| --- | --- | --- | --- | --- | --- | --- | --- |
|  |  | Left | Right | Left | Right | Left | Right |
| **1** | 2.99 (0.87) | 0.19 | 0.12 | 0.13 | 0.42 | -0.06 | 0.32 |
| **1.5** | 4.6 (1.6) | 0.27 | 0.18 | 0.18 | **0.37*** | -0.11 | 0.21 |
| **2** | 5.62 (1.64) | **0.33*** | 0.24 | 0.22 | **0.36*** | -0.13 | 0.15 |
| **2.5** | 7.03 (1.84) | 0.31 | 0.26 | 0.32 | 0.31 | 0 | 0.1 |
| **3** | 8.71 (2.17) | 0.31 | 0.31 | **0.33*** | **0.36*** | 0.01 | 0.1 |
| **3.5** | 7.64 (2.2) | **0.34*** | **0.4*** | 0.29 | **0.46**** | -0.08 | 0.14 |
| **4** | 5.44 (2.26) | **0.44*** | **0.45*** | 0.29 | **0.53**** | -0.2 | 0.16 |
| **5** | 3.56 (2.27) | **0.46**** | **0.44*** | 0.3 | **0.57**** | -0.21 | 0.21 |
| **7** | 1.06 (2.16) | **0.43*** | **0.45*** | 0.21 | **0.54**** | -0.26 | 0.17 |

**Supplementary Table 4.** Mean and standard error of metabolite concentrations in the hand region of the motor cortex in ALS patients at baseline and 6-month follow-up. *Indicates significance at p<0.05.

|  | **Metabolite** | **Baseline** | **6-Month** | **P-value** |
| --- | --- | --- | --- | --- |
| **ALS** |  |  |  |  |
| **(n=10)** | NAA/Cr |  |  |  |
|  | Left | 1.39 (0.03) | 1.35 (0.03) | **0.03*** |
|  | Right | 1.34 (0.03) | 1.33 (0.04) | 0.65 |
|  | Glx/Cr |  |  |  |
|  | Left | 1.6 (0.04) | 1.65 (0.04) | 0.14 |
|  | Right | 1.72 (0.06) | 1.71 (0.05) | 0.75 |
|  | Glx/NAA |  |  |  |
|  | Left | 1.15 (0.03) | 1.23 (0.03) | **0.02*** |
|  | Right | 1.29 (0.03) | 1.29 (0.04) | 0.89 |

**Supplementary Table 5.** Mean and standard error of cortical thickness of the Arm region of the primary motor cortex (BA4) in ALS patients and healthy controls. ALS patients were stratified based on severity of UMN dysfunction as defined by percent change in short interval intracortical inhibition (SICI).

|  | **Average Cortical Thickness (mm)** | | | |
| --- | --- | --- | --- | --- |
|  | **Abnormal SICI**  **(n=20)** | **Normal SICI**  **(n=14)** | **Control**  **(n=20)** | **P-value** |
| **Left Hemisphere** | 2.42 (0.05) | 2.33 (0.06) | 2.39 (0.06) | 0.46 |
| **Right Hemisphere** | 2.31 (0.05) | 2.25 (0.06) | 2.29 (0.06) | 0.62 |

**Supplementary Table 6.** The sensitivity and specificity of NAA and Glx/NAA metabolite concentrations in the hand region of the left motor cortex were assessed using ROC curves. Sensitivity and specificity of metabolite change was calculated between healthy controls and the ALS cohort. Patients were stratified based on severity of upper motor neuron dysfunction as defined by percent change in short interval intracortical inhibition (SICI). Likelihood ratio (LR) is defined as the probability of having the disease. An LR value>1 indicates that the probability of having the target disease is increased, relative to absence of disease.

|  | **Metabolite** | **Value** | **Sensitivity (%)** | **Specificity (%)** | **LR** |
| --- | --- | --- | --- | --- | --- |
| **ALS (Combined)** | NAA | <1.3 | 32 | 94 | 4.7 |
|  |  | <1.4 | 59 | 75 | 2.4 |
|  |  | <1.5 | 88 | 31 | 1.3 |
|  | Glx/NAA | >1.1 | 91 | 38 | 1.5 |
|  |  | >1.15 | 71 | 75 | 2.8 |
|  |  | >1.2 | 53 | 88 | 4.2 |
|  |  | >1.25 | 32 | 88 | 2.6 |
| **ALS (Low SICI)** | NAA | <1.3 | 50 | 94 | 8 |
|  |  | <1.4 | 75 | 75 | 3 |
|  |  | <1.5 | 95 | 31 | 1.4 |
|  | Glx/NAA | >1.1 | 76 | 44 | 1.7 |
|  |  | >1.15 | 80 | 75 | 3.2 |
|  |  | >1.2 | 70 | 88 | 5.6 |
|  |  | >1.25 | 50 | 88 | 4 |
| **ALS (High SICI)** | NAA | <1.3 | 7 | 94 | 1.1 |
|  |  | <1.4 | 36 | 81 | 1.9 |
|  |  | <1.5 | 79 | 13 | 1.1 |
|  | Glx/NAA | >1.1 | 86 | 38 | 1.4 |
|  |  | >1.15 | 57 | 56 | 1.3 |
|  |  | >1.2 | 29 | 88 | 2.3 |
|  |  | >1.25 | 0 | 88 | 0 |

**Supplementary Figure 1.** Metabolite concentrations in the hand region of the motor cortex in 26 ALS patients and 20 healthy control participants, excluding patients not receiving Riluzole at the time of assessment. ALS patients were stratified based on severity of upper motor neuron dysfunction as defined by percent change in short interval intracortical inhibition (SICI). Median and interquartile range are shown. Group differences were assessed using ANCOVA, covarying for age and gender. *Indicates significance at p<0.05; ** p<0.01. Note: ^1^H-MRS of the right hemisphere was not acquired for 1 ALS patient in the high (H-SICI) and low (L-SICI) SICI cohorts, respectively.

**Supplementary Figure 2.** Metabolite concentrations in the hand region of the motor cortex in 34 ALS patients and 20 healthy control participants. Median and interquartile range are shown. Group differences were assessed using ANCOVA, covarying for age and gender. *Indicates significance at p<0.05; ** p<0.01. Note: ^1^H-MRS of the right hemisphere was not acquired for 2 ALS patients.
